# TME1/339: Telematic Applications in Ophthalmology: Transfer of Angiographic Images over the Internet

**DOI:** 10.2196/jmir.1.suppl1.e108

**Published:** 1999-09-19

**Authors:** K Wörle

**Affiliations:** ^1^Augenklinik Universität Regensburg, RegensburgGermany

**Keywords:** Telemedicine, Ophthalmology, Angiography, Retina, Wavelet compression

## Abstract

**Introduction:**

Many methods of advanced Medical Imaging are only available at larger hospital-based imaging departments. Nevertheless, medical care would benefit if those diagnostic images could easily be transferred to doctors in private practices, where patients receive further treatment and monitoring of their diseases. Crucial to the acceptance and continuous operation of telematic applications, which are intended for a wider use in primary care, are criteria like simplicity, economic efficiency and user-friendliness.

**Methods:**

In the case of Ophthalmology, angiographic images of the retina are of high diagnostic value. Typically, digitised angiography series require several megabytes of storage, which in turn would lead to unacceptably long download times when transferring these sets of images over dial-up connections. Thus, appropriate compression techniques have to be used.

**Results and Discussion:**

Wavelet encoding proves to be very efficient for compressing angiography data. High compression rates are achieved at an acceptable level of image deterioration. In addition, commercial tools are available to automate the process of image compression. For decoding an appropriate browser plug-in is freely distributed, thus standard Internet technology and protocols can be used for data transfer. Furthermore, the coding and decoding process can be password protected to ensure an enhanced level of security. In our Department we set up a procedure to transfer and display angiographic and other ophthalmic images, where we are using only standard software components, which are commonly used for Internet access. Therefore no special skills or training is necessary for the users. Data compression is performed, automatically, on both the sender and recipient side, with software that seamlessly integrates into the transfer process. This configuration proves to be easy to use, versatile, and cost effective.

